# Motion Capture and Dance Image Discrimination Based on Silent Positioning Algorithm

**DOI:** 10.1155/2022/1187548

**Published:** 2022-06-13

**Authors:** Lin Zhu

**Affiliations:** College of Arts, Wuhan Sports University, Hubei, Wuhan 430079, China

## Abstract

The silent location algorithm of the sensor network is a time synchronization location algorithm that can serve multiple users. Based on the detailed analysis of the development status of human motion measurement technology, this paper proposes a design of human motion capture device based on sensor network and realizes data conversion and filtering through experimental design. In this experiment, the sensor data processing is used to capture the human body positioning information in real time. The established node does not need to make a sound during the whole process and, at the same time, has strong concealment and easy expansion. In the silent positioning algorithm for sensor network nodes, combined with the sound ray tracking technology, the correction parameters are controlled according to the change of the objective function, and the effectiveness of the method is verified by simulation analysis. In terms of motion capture, the system can monitor, detect, and record human motion in real time. In order to obtain more high-resolution dance images with high-definition details and correspondingly process the dance images to improve the resolution, this paper uses the silent positioning algorithm technology to study the image resolution, and this is of great help to improve the resolution.

## 1. Introduction

Through experimental design, the sensor data is converted and filtered, and the human body motion information is extracted, and the sensor chip error that may exist in the sensor node is analyzed, such as the vibration and variance of the acceleration sensor data, the cumulative error of the gyroscope, and the accelerometer errors and the sensitivity of the geomagnetic sensor to the surrounding environment [1]. On this basis, it focuses on the solutions to the preprocessing errors of various sensor chips, describes the data stream header of the device node in detail, and configures the data frame and controller to ensure the correct capture and transmission of sensor data [2]. Usually consider sensor network positioning, but the node to be found has to repeatedly communicate with the locomotive node. If you want to find multiple nodes, the location service or the node to be found must be muted at the same time due to operational needs [3]. In this case, a silent location algorithm is proposed for the first time. The algorithm does not need to synchronize clocks between nodes and needs to find the nodes may be in silent reception time during the whole process [4]. However, the algorithm ignores the inhomogeneity of the sound velocity distribution. In order to find a way to reduce the order, the algorithm solves the problem of node positioning, which is a problem in blind spot positioning [5]. Although subsequent scientists have made great efforts for silent positioning, it did not solve the original problem. It has a certain impact on the uneven sound velocity distribution of the UPS algorithm, and it has not been fundamentally solved. Aiming at the complexity of dance learning, a dance teaching method based on motion capture technology is proposed [6]. Using motion capture technology, the entire complex dance movement is broken down into multiple small segments, and teaching animations are made, which can guide students to follow the small dance movements [7]. Fragments for learning and imitation make abstract theories vivid, intuitive, and easy to understand and encourage innovation in education and teaching methods [8]. Motion capture technology is a technology that has developed rapidly in the past two to three decades. Many researchers at home and abroad have invested a lot of scientific research strength in this field, made scientific progress, and obtained rich theoretical support in various aspects such as the way and implementation of the proposal [9]. This article mainly combines motion capture technology and dance teaching mode and provides an efficient data processing method and implementation method. The effectiveness and feasibility of this method are verified by a large amount of experimental data, which is of great significance to the realization of digital dance teaching. The main characteristics of dance image resolution technology are the fast speed of obtaining information, less access to various data, a wide range of access to all data and dance image data, and various acquisition methods [10]. People are increasingly demanding the accuracy of dance images. High-resolution dance images contain more detailed information [11]. They can also provide people with good visual effects and provide useful information for further data processing. This kind of dance image processing technology has attracted more and more attention, and the superresolution of dance images has been vigorously developed [12].

## 2. Related Work

The literature proposes an active capture device that captures the process of human activity in real time and tests its effect and performance in capturing the movement of a single sensor node [13]. The results show that the sensor node has excellent performance and can be used to capture human activities and then, through experimental design, capture human motion, design a program to record human motion, read data output from DLL, calculate and manage virtual human model, and realize human body Real-time motion capture of upper limbs [14]. The literature analyzes the requirements of sensor nodes for the position of the human body, and through the aggregation of sensor data in the human motion measurement test, the quaternion representation of sensor data is given. According to the sensor data, the virtual computer feature model is activated and calibrated, and it is driven in real time during the movement process to realize the real-time recording of the human body movement. Aiming at the shortcomings of the existing UPS algorithm, the literature proposes a new sensor network positioning algorithm, which uses the sound beam tracking technology to solve the problem of uneven sound velocity distribution and uses the Gauss-Newton method to evaluate the optimal position node for positioning [15]. The problem of positioning the UPS algorithm in the dark area is solved. In order to solve the problem of poor array positioning, the literature proposed an improved Tikhonov regularization method [16]. According to the iterative effect, feedback control is performed on the regularization parameters to improve the reliability of the algorithm. Finally, through simulation analysis, the needs of each step and the impact of each error on the performance of the algorithm are discussed, and the effectiveness of the algorithm is verified. In order to meet the needs of human beings for high-efficiency and high-precision position detection methods, it is recommended to adopt an effective position analysis method based on the similarity between recognition layers [17]. Through the effective extraction of human skeleton and plane, an effective matching mechanism based on plane feature vectors and angles is established, that is, combined with dance teaching. Through experimental tests, not only can the human body posture be analyzed stably and accurately, but also the differences in human body movements can be actually obtained, providing good theoretical support for dancers through scientific dance training. The literature first uses the optical motion capture system to obtain the dancer's motion parameters in real time, obtain the human bone data, establish the human motion model, and complete the establishment of the motion model database; secondly, use the function of the plane similarity method to coordinate the motion position, and finally, apply the method. Based on the analysis of precise dance postures, combined with dance teaching, the standardized and continuous teaching activities have been studied. Experimental data shows that this method is a very good standardized analysis method of human posture, and it is a very important dance intelligence, scientific teaching. At the same time, it also laid a good foundation for the next step of the backtracking of dance movement data. The literature combines collection technology and teaching to make up for the shortcomings of traditional teaching methods. It has advantages in acquiring and transferring skills that traditional teaching does not have. Then, according to the three-dimensional analysis results of the flow data, it is compared with the standard operating position in a timely manner. The standardization of adjustment activities has significantly improved students' learning and teachers' teaching efficiency. The literature obtains some information about the outside world through dance images. In order to obtain more adequate image information, the resolution of snapshots is particularly important. People's requirements for the resolution of snapshots have also been greatly improved, so we continue to explore different methods to further improve Snapshot resolution, and superresolution reconstruction dance is an effective method.

## 3. Sensor Network Silent Positioning Algorithm

The sensor coordinate system should be used to indicate the size and direction of the corresponding alarm information collected by the sensor in each direction. It is usually defined in the design of the chip. In general, it corresponds to the right hand of the Cartesian coordinate system. In this article, the sensor node's the hardware design is always consistent with the two chip directions selected in the PCB design. Therefore, the sensor chip coordinate system can also be regarded as the sensor node coordinate system, allowing the motion sensor to be taken over.

The human body coordinate system refers to the human body coordinate system using sensor nodes. Its function is to map the virtual three-dimensional space relationship between the model of the human body sensor coordinate system and the Earth coordinate system and the computer through the sensor data. In order to facilitate the calculation, the coordinate system of each human body should be consistent with the direction of the corresponding sensor node coordinate system. For example, in the head coordinate system, taking the center of the human head as the starting point of coordinates, the head is viewed horizontally, the *X* axis is horizontally facing forward, the *Y* axis is horizontal to the left shoulder, and the *Z* axis is perpendicular to the horizontal plane and points to the top of the head. The *X* axis is parallel to the plane of the back of the hand and points to the finger, and the *Y* axis is parallel to the back of the hand that points to the thumb. The *Y* axis is parallel to the back of the hand and points to the thumb. Other coordinates are similar.

Coordinate conversion is the conversion of coordinates from one coordinate area to another, which usually includes coordinate rotation and coordinate translation. Calculate the coordinates of the rotated point. Coordinate transformation is added to the coordinates of the converter itself. Therefore, the coordinate conversion is shown in the following formula:

(1) $$\begin{array}{c} \left[\begin{array}{c} X\\ Y\\ Z \end{array}\right]=\left[R\right]\left[\begin{array}{c} \begin{array}{c} X_{O}\\ Y_{O} \end{array}\\ Z_{O} \end{array}\right]+\left[T\right]. \end{array}$$

The conversion between the limb joints of the virtual human body model is also carried out according to the above formula, and the offset vector is the actual three-dimensional parameter of the virtual model coordinate system. The component on the coordinate axis is integrated with the acceleration value.

According to the overall design of this article, it is necessary to use three sensor nodes representing the upper part of the human body according to the hands, forearms, and forearms. The torque matrix between any sensor node coordinate system and the limb coordinate system is the wear point. The specific method is as follows:

(1) The upper limb is parallel to the horizontal plane, and the palm is down; the static position allows the weight acceleration of the forearm sensor node and the armrest sensor node to be calibrated, so as to calibrate the RF and RH of the calibratable hand rotation matrix as in formulas (2) and (3) as follows:
  (2) $$\begin{array}{c} Y_{U}=-\frac{g_{U}^{M}}{\left|g_{U}^{M}\right|}, \end{array}$$
  (3) $$\begin{array}{c} Y_{F}=-\frac{g_{F}^{M}}{\left|g_{F}^{M}\right|}. \end{array}$$
(2) According to the previous position, the upper part is rotated by about 180° from the back of the body, and then by about 180°; starting from the dynamic position, the angular velocity output of the large hand, small hand, and hand position sensor nodes during the rotation of the upper limb are shown in formulas (4) and (5):
  (4) $$\begin{array}{c} X_{F}=-\frac{\omega_{F}^{S}}{\left|\omega_{F}^{S}\right|}=\frac{\omega_{F}^{P}}{\left|\omega_{F}^{P}\right|}, \end{array}$$
  (5) $$\begin{array}{c} X_{H}=-\frac{\omega_{H}^{S}}{\left|\omega_{H}^{S}\right|}=\frac{\omega_{H}^{P}}{\left|\omega_{H}^{P}\right|}. \end{array}$$
At the same time, RF and RH can be obtained as shown in formulas (6) and (7):
  (6) $$\begin{array}{c} R_{F}=\left(X_{F},X_{F}\times \left(Y_{F}\times X_{F}\right),Y_{F}\times X_{F}\right), \end{array}$$
  (7) $$\begin{array}{c} R_{H}=\left(X_{H},X_{H}\times \left(Y_{H}\times X_{H}\right),Y_{H}\times X_{H}\right). \end{array}$$
(3) Then, lift the upper limb at a constant speed until the upper limb line is perpendicular to the trunk line, and the palm surface is perpendicular to the horizontal plane. Then Ru can calibrate the rotation matrix as shown in the following formula:
  (8) $$\begin{array}{c} Y_{U}=\frac{\omega_{U}}{\left|\omega_{U}\right|}. \end{array}$$
(4) The upper limbs descend naturally, and then the upper limbs are lifted from the side at a steady speed until a straight line of the upper limbs is perpendicular to a straight line of the torso, and the palms face down. At this time, the static recovery is as shown in formulas (9) and (10):

(9) $$\begin{array}{c} Z_{U}=-\frac{g_{U}^{V}\times g_{U}^{H}}{\left|g_{U}^{V}\times g_{U}^{H}\right|}, \end{array}$$

(10) $$\begin{array}{c} R_{U}=\left(Z_{U}\times Y_{U},Y_{U},Y_{U}\times \left(Z_{U}\times Y_{U}\right)\right). \end{array}$$

The rotation matrix between the *i* th limb and the *i* + 1 th limb in the rigid limbs of the human body is shown in the following formula:

(11) $$\begin{array}{c} R_{i}^{i+1}={\left(R_{i+1}\right)}^{-1}{\left(R_{i+1}\right)}^{-1}R_{i}R_{i}^{E}. \end{array}$$

The transformation matrix from the *i* th limb to the *i* + 1 th limb in the rigid limbs of the human body is shown in the following formula:

(12) $$\begin{array}{c} P_{i}^{i+1}=\left[\begin{array}{cc} R_{i}^{i+1} & T_{i}^{i+1}\\ 0 & 1 \end{array}\right]. \end{array}$$

According to the multiplication of the transformation matrix, the transformation of the first limb relative to the i-th limb can be obtained as shown in the following formula:

(13) $$\begin{array}{c} P_{1}^{i}=P_{i-1}^{i}-P_{i-2}^{i-1}\cdots P_{2}^{3}P_{1}^{2}. \end{array}$$

In this way, the position of the first node of the motion sensor and the real-time position and coordinates of the i-th limb can be calculated. Therefore, it is possible to calculate the real-time position and action mode of each rigid limb of the human body by sequentially calculating all the rigid limbs connected by joint rotation; understand the virtual character model of the computer to simulate the real human body.

From this, the rotation quaternion information of each node arranged in the upper limb relative to the previous node can be calculated. The calculation method is as shown in formulas (14), (15), and (16) as follows:

(14) $$\begin{array}{c} q_{12}=q_{B}⊗q_{2}⊗q_{1}⊗q_{A}, \end{array}$$

(15) $$\begin{array}{c} q_{23}=q_{C}⊗q_{3}⊗q_{2}⊗q_{B}, \end{array}$$

(16) $$\begin{array}{c} q_{34}=q_{D}⊗q_{4}⊗q_{3}⊗q_{C}. \end{array}$$

The angle of front and back rotation (front and back direction) of the shoulder and the angle of outward expansion and inward contraction (left and right direction) are shown in formulas (17) and (18):

(17) $$\begin{array}{c} \phi_{1}=a\tan2\left(-2q_{12}\left(2\right)q_{12}\left(4\right)-2q_{12}\left(1\right)q_{12}\left(3\right),q_{12}^{2}\left(1\right)+q_{12}^{2}\left(2\right)-q_{12}^{2}\left(3\right)-q_{12}^{2}\left(4\right)\right), \end{array}$$

(18) $$\begin{array}{c} \phi_{2}=a\tan2\left(-2q_{12}\left(3\right)q_{12}\left(4\right)+2q_{12}\left(1\right)q_{12}\left(2\right)q_{12}^{2}\left(1\right)-q_{12}^{2}\left(2\right)+q_{12}^{2}\left(3\right)-q_{12}^{2}\left(4\right)\right). \end{array}$$

The front and back rotation angle of the forearm Φ_5_, the wrist contraction and extension angle Φ_6_, and the wrist extension and flexion Φ_7_ are as shown in formulas (19), (20), and (21):

(19) $$\begin{array}{c} \phi_{5}=a\tan2\left(-2q_{34}\left(3\right)q_{34}\left(4\right)-2q_{34}\left(1\right)q_{34}\left(2\right)q_{34}^{2}\left(1\right)-q_{34}^{2}\left(2\right)+q_{34}^{2}\left(3\right)-q_{34}^{2}\left(4\right)\right), \end{array}$$

(20) $$\begin{array}{c} \phi_{6}=a\sin\left(2q_{34}\left(2\right)q_{34}\left(3\right)+2q_{34}\left(1\right)q_{34}\left(4\right)\right), \end{array}$$

(21) $$\begin{array}{c} \phi_{7}=a\tan2\left(-2q_{34}\left(2\right)q_{34}\left(4\right)+2q_{34}\left(1\right)q_{34}\left(3\right),q_{34}^{2}\left(1\right)+q_{34}^{2}\left(2\right)-q_{34}^{2}\left(3\right)-q_{34}^{2}\left(4\right)\right). \end{array}$$

At the same time, the position of the palm center in the virtual character model can be obtained by the following formula:

(22) $$\begin{array}{c} \text{XYZ}=\sum_{i=1}^{3}v_{i}^{\text{rot}}. \end{array}$$

Among them, XYZ represents the coordinates of the center of the palm in the model, and its vector modulus is equal to the length of the corresponding limb. The relationship between them is shown in formulas (23), (24), and (25):

(23) $$\begin{array}{c} v_{1}^{\text{rot}}=q_{12}^{*}⊗v_{1}⊗q_{12}, \end{array}$$

(24) $$\begin{array}{c} v_{2}^{\text{rot}}={\left(q_{23}⊗q_{12}\right)}^{*}⊗v_{2}⊗\left(q_{23}⊗q_{12}\right), \end{array}$$

(25) $$\begin{array}{c} v_{3}^{\text{rot}}={\left(q_{34}⊗q_{23}⊗q_{12}\right)}^{*}⊗v_{3}⊗\left(q_{34}⊗q_{23}⊗q_{12}\right). \end{array}$$

## 4. Dance Motion Capture Method and Dance Image Discrimination and Reconstruction

### 4.1. Dance Motion Capture Method

The mobility capture system should be used to analyze the mobility position of the person and evaluate the mobility characteristics of the person from different angles. This article uses the basic characteristics of movement to represent the main parts of the human body. The straight lines between the key points represent rigid bodies, so their shape never changes. Mobile sensory analysis is the process of monitoring, capturing, acquiring, and analyzing human body position characteristics to obtain appropriate motion parameters. Through the effective combination of motion analysis and teaching, the learning system is more personalized. It can also break down the performance standards of the performers through detailed and progressive demonstrations of each dance activity. The obtained parameters are conducive to the quantitative analysis of dance movement postures and provide beneficial help for the scientific and intelligent dance teaching.

**Figure 1:**
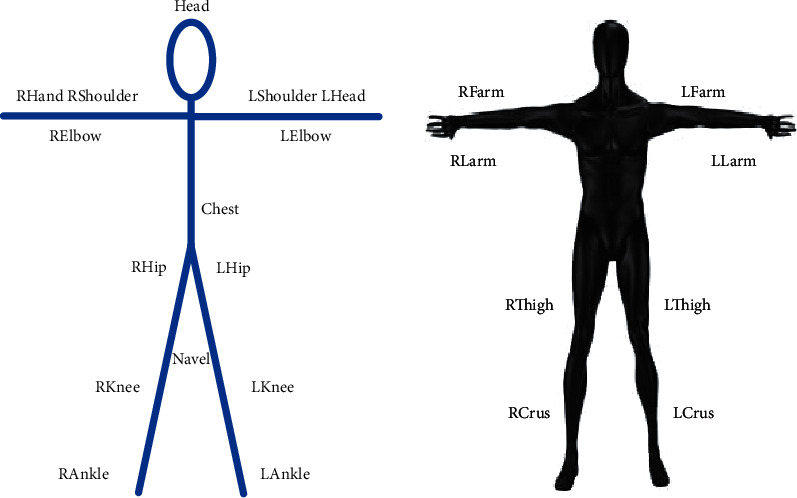
Human skeleton and model diagram.

Human movement is a complex process. A person's movement can take a simple chain movement, connecting some rigid organs to weigh muscles, nerves, etc., as shown in Figure 1. The upper limbs are composed of two stiff bodies, a large arm and a forearm connected to the elbow; the lower limbs are composed of a thigh and a leg related to the hip joint. The thigh and tibia are connected to the knee joint. The head, body, and buttocks also have a series of things in common.

**Figure 2:**
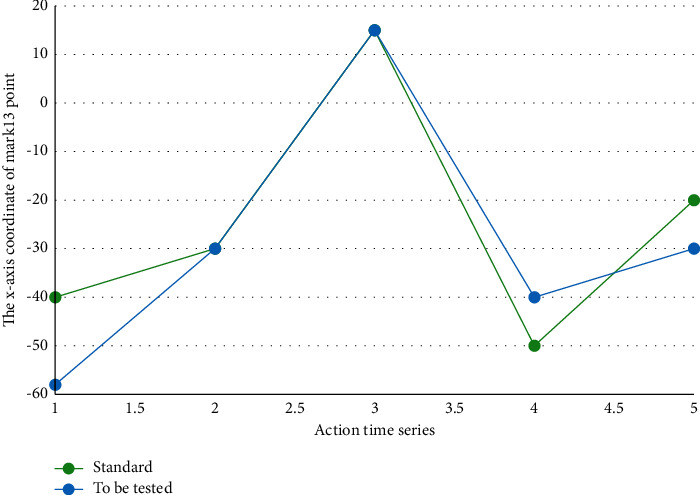
Comparison of motion trajectories in a single direction between a single identification point.

If the difference is less than the threshold, the trainer determines the threshold, and the two recognition points are considered similar. If the difference is greater than the threshold, the two identification points are not similar. The trajectory comparison of the same marking point of the standard action and the action to be measured is shown in Figure 2.

The traditional Euclidean distance direct comparison method compares the trajectories of two moving targets. By coordinating each axis, the corresponding distance difference is obtained, and the data coincidence degree is calculated according to the preset threshold. However, this method not only is computationally expensive, but also depends on the characteristics of the measured object. If the body parts of the object, such as height, being fat, and being thin, change, the distance between the recognition points will also change, so it must be remeasured and calculated. Therefore, due to the strict requirements for moving objects, the use of traditional methods is restricted, which not only greatly reduce the effectiveness of activities, but they are not universal as well.

According to the standardization of dance movement requirements and the relative range of movement of the human skeleton, the feature vectors of the main movement parts of the human body are specified as shown in Table 1.

According to the standardization of dance movement requirements and the relative range of movement of the human skeleton, the angles of the main movement parts of the human body are specified as shown in Table 2:

#### 4.1.1. Limbs

The movement direction of the limbs can be determined by the inner product of the characteristic plane of the limbs, the normal vector, and the vertical strain vector, and the normal movement of the limbs can be accurately measured at this angle.

#### 4.1.2. Head

The normal vector V_5_ of the head feature plane P_5_ is compared with the vertical standing direction *V*_stand_ to determine the head movement angle *θ*_5_ of the motion model. When the human body looks straight ahead, the V_5_ and *V*_stand_ directions are parallel.

#### 4.1.3. Torso

The torso includes chest and hips, mainly rotating movement and waist bending movement, among which the rotation movement compares the spine direction vector V_6_ and the vertical standing direction *V*_stand_ to transform the angle *θ*_6_. For the bending motion, when the human body stands upright, the hip plane P_7_ remains horizontal, and its plane normal vector V_7_ is parallel to the vertical direction *V*_stand_.

The angle between the edge vector of the characteristic plane and the vertical direction is used to judge the relationship between the limbs and the trunk joints, and the angle between the joints is judged, as shown in Table 3.

This article uses cosine similarity as a function of similarity. This method can not only measure the difference between vectors, but also measure the similarity and difference between angles. According to the cohesion value of the product of two vectors in cosmic space, the difference between two vectors can be measured.

Taking the movement of the left arm of a dance trainer as an example, taking the characteristic plane P1 of the left arm as the basic calculation plane, three discriminant parameters {Sim(*V*_1_, *V*_Stand_), Corr(*θ*_1_), Corr(*θ*_8_)} can be obtained through the above three. These parameters are the basis for discrimination to determine the overall motion posture of the left arm. The experiment proves that the calculation error is effectively reduced, and the results are shown in Table 4.

**Figure 3:**
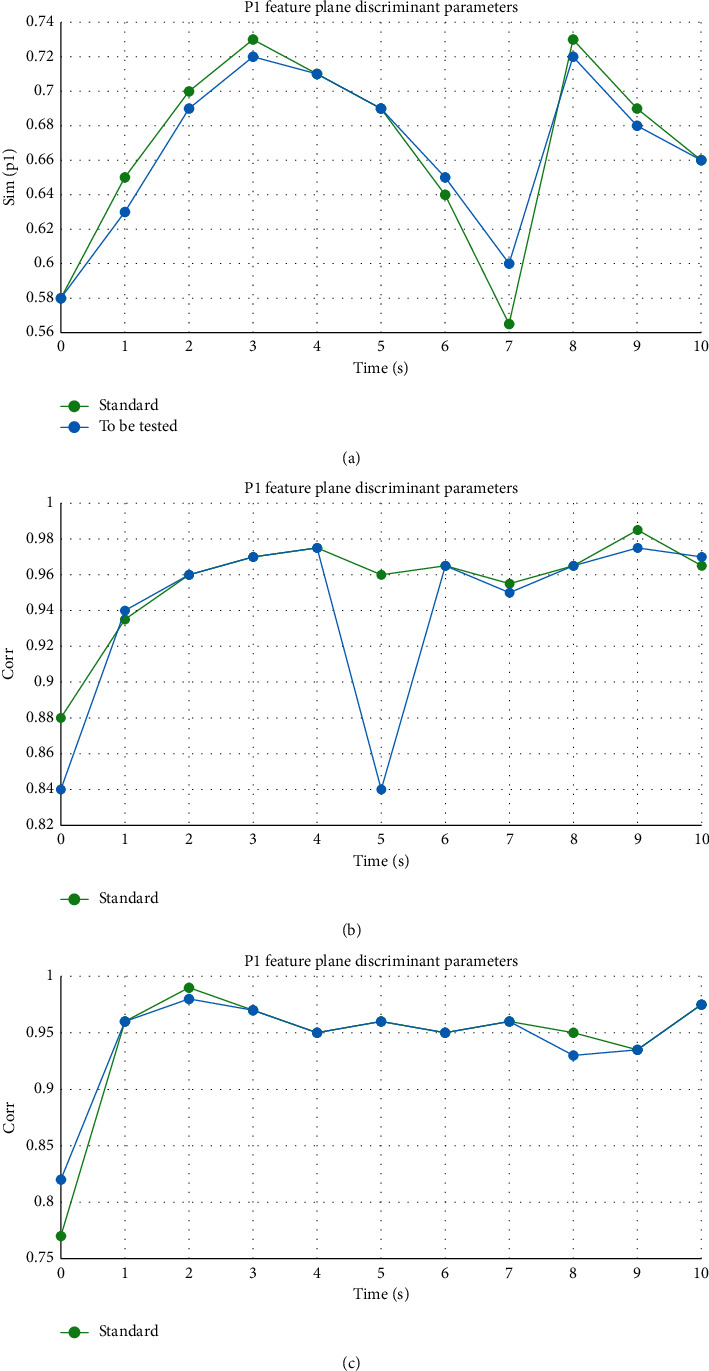
Timing diagram of left arm motion related parameters (0–10 s). (a) The difference in the direction of movement of the left arm. (b) Difference in movement of left arm joint angle. (c) The difference in the angle between the left arm and the trunk.

Through the comparative analysis of the degree of difference, the standard measurement values of the 4–6 s time period and the 6–8 s time period are obtained. There is a significant difference between the degree of elbow joint bending of the subjects and the swing amplitude of the left hand. The left-hand movement comparison chart is shown in Figure 3, showing the difference between the test operation and the standard operation. By comparing the experimental results, the method can clearly and effectively identify the differences and standardization between moving objects and has a high resilience, which lays a foundation for the training of scientific dance.

In the dance teaching process, use motion capture technology to realize intelligent teaching, improve teaching level, update teaching methods, solve problems through the retrospective and time-effective teaching of dance teaching, and analyze and playback the recorded motion data, which is effective. It solves the problem of repeated explanations and demonstrations in the classroom by teachers.

### 4.2. Discrimination and Reconstruction of Dance Images

#### 4.2.1. Dance Image Reconstruction Model and Optimization

Markov randomly deployed a superresolution reconstructed image. In the training stage, the training is used to determine the images to be established in the database, and the Markov network is used to simulate the low-resolution dance image blocks and high-resolution image blocks in the database. Because the main difference between low-resolution and high-resolution dance images lies in high-frequency information, that is, texture, the texture of dance images is examined by studying texture. Use two-digit interpolation to increase color information and dance image structure. Connect the reconstructed dance image with the two-level interpolation image to get the final result. Practice has proved that the reconstruction quality of dance images can achieve good results in both subjective visual effects and objective indicators.

**Figure 4:**
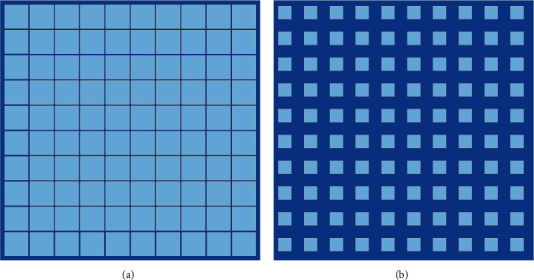
Image blocks in training. (a) IF image block. (b) HF image block.

At this stage, we need to build a database based on the medium and high-frequency dance picture information obtained by preprocessing before and randomly select a dance picture block from the image database. In the training process, in a database, we need to overlap the training data to obtain a sample as shown in Figure 4.

#### 4.2.2. Improved Implementation of Superresolution Reconstruction of Random Field Remote Sensing Images

The original method is based on reconstructing Freeman's learning. This method is mainly to find the most similar low-resolution image blocks in the training library, evaluate the high-frequency details of the low-resolution dance images, and occupy a larger resolution part in the low-resolution images. This method has the following errors: the first point is that the image block contains more information, and it takes longer to search for similar images; the second point is that the input of the dance image and the training set is not relevant, and the reconstruction image effect is poor; third, the reconstruction method of this article is to divide the dance picture into a structural part and a texture part. There is a big difference in texture between high-resolution snapshots and low-resolution snapshots. First of all, the texture of the dance picture has been restored. In order to avoid the lack of color information in the training package, it is necessary to restore the gray information dance image. Secondly, perform degrading processing on the dance image. After converting the reconstructed training image YCbCr, select the gray component of the dance image for full degradation processing based on the L2 norm, obtain the structure and texture information of the snapshot, and then select training. The high-resolution block texture part of the high-resolution snapshot in the package interpolates the texture part of the input dance image to obtain a low-resolution snapshot. This paper reconstructs the resolution of low dance images and then studies texture images. Nonlocal similarity and sustainability are two good methods for obtaining prior information. In recent years, reconciling low-order matrices is a way to approximate low-order matrices. In fact, the noise set in the matching image block is removed by a low-order matrix. According to the latest results of random matrix theory, an asymptomatic matrix reconstruction model is used to solve this problem.

## 5. Conclusion

Based on analyzing the characteristics of the sensor network, a human motion capture device based on the human sensor network is developed. To capture and transmit the movement information of the important links in the human body's movement process, the computer program combines the sensor data captured by the device to guide the establishment of the virtual three-dimensional virtual character model in real time. This article analyzes and introduces the application fields of activity capture technology, points out the value and development space of activity capture products, and analyzes the advantages of using sensors. On this basis, this article analyzes the design ideas and requirements and proposes an overall design plan. In order to solve the problem of dark areas, several solutions have been proposed, ignoring the impact of uneven sound velocity and providing an advanced positioning method for human sensor networks. This algorithm combines the sound beam tracking technology with the Gauss-Newton iteration method, which can obtain a unique optimal location node, which is located at any position in the communication area of the radio beacon array and improves the impact, and the Tikhonov adjustment method with feedback control can maintain the good positioning performance of the algorithm even if the beacon array is not good. Simulation results show that the algorithm has better performance than the existing UPS algorithm, and it can still maintain a good positioning effect when there is a certain error in the input parameters. At the same time, the algorithm has a certain degree of certainty in the use of beacons. Due to the development of society, the development of science and technology, and the improvement of computer software and hardware, many scientists have decided to conduct scientific research and further teaching and training through computers. Motion capture technology is a new scientific research topic that has emerged in recent years. This article analyzes the posture of human motion, proposes a position analysis method based on vector matching functions, and analyzes the position characteristics of human motion. At the same time, combined with college physical education, it analyzes the development prospects of capture technology in sports dance teaching and the importance of implementing active research, which provides an effective theoretical basis for scientific training. This article proposes a dance based on the suitability of object vectors. The posture analysis method can accurately analyze the dance position of the human body, realize the significant difference in the human movement ability, and provide theoretical support for scientific dance training. As one of the country's important strategic technologies, dance image resolution technology has been vigorously developed, and dance image resource technology is widely used in resource identification. Therefore, the accuracy of dance images is improving.

## Figures and Tables

**Table 1:** Discriminant vector on the feature plane.

Feature plane	Feature vector
Left arm (P_1_)	*V*_*I*_=*V*_LLarm_ × *V*_LFarm_
Right arm (P_2_)	*V*_2_=*V*_RLarm_ × *V*_RFarm_
Left leg (P_3_)	*V*_3_=*V*_LThigh_ × *V*_LCrus_
Right leg (P_4_)	*V*_4_=*V*_RThigh_ × *V*_RCrus_
Head (P_5_)	*V*_5_=*V*_LHead_ × *V*_RHead_
Chest (P_6_)	*V*_6_=*V*_LChest_ × *V*_RChest_
Hip (P_7_)	*V*_7_=*V*_LHip_ × *V*_RHip_

**Table 2:** Discriminate the included angle on the characteristic plane.

Feature plane	Geometric relationship	*θ*_Max_	*θ*_Min_
Left elbow (P1)	*θ*_1_=*V*_LFarm_, *V*_LLarm_	180°	40°
Right elbow (P2)	*θ*_2_=*V*_RFarm_, *V*_RLarm_	180°	40°
Left knee (P3)	*θ*_3_=*V*_LThigh_, *V*_LCrus_	180°	35°
Right knee (P4)	*θ*_4_=*V*_RThigh_, *V*_RCrus_	180°	35°
Head (P5)	*θ*_5_=*V*_RHead_, *V*_Lhead_	—	—
Chest (P6)	*θ*_6_=*V*_RChest_, *V*_LChest_	—	—
Hip (P7)	*θ*_7_=*V*_RHip_, *V*_LHip_	—	—

**Table 3:** Discrimination angle of joint connection.

	Geometric relationship	*θ*_Max_	*θ*_Min_
Left shoulder	*θ*_8_=*V*_LFarm_, *V*_Stand_	180°	0°
Right shoulder	*θ*_9_=*V*_RFarm_, *V*_Stand_	180°	0°
Left hip	*θ*_10_=*V*_LThigh_, *V*_Stand_	45°	0°
Right hip	*θ*_11_=*V*_RThigh_, *V*_Stand_	45°	0°
Head	*θ*_12_=*V*_5_, *V*_Stand_	45°	0°

**Table 4:** Related parameters of left arm action posture (0–1 s).

Test subject	sim(*V*_1_, *V*_Stand_)	Corr(*θ*_1_)	Corr(*θ*_8_)
Standard object	0.5263	0.8658	1.0141
Object to be tested	0.6522	0.7546	0.8659

## Data Availability

The data used to support the findings of this study are available from the corresponding author upon request.
